# Exploring theophylline-1,2,4-triazole tethered *N*-phenylacetamide derivatives as antimicrobial agents: unraveling mechanisms via structure-activity relationship, *in vitro* validation, and *in silico* insights

**DOI:** 10.3389/fchem.2024.1372378

**Published:** 2024-04-05

**Authors:** Sadaf Saeed, Irum Shahzadi, Ameer Fawad Zahoor, Aamal A. Al-Mutairi, Shagufta Kamal, Shah Faisal, Ali Irfan, Sami A. Al-Hussain, Muhammed Tilahun Muhammed, Magdi E. A. Zaki

**Affiliations:** ^1^ Department of Chemistry, Government College University Faisalabad, Faisalabad, Pakistan; ^2^ Department of Chemistry, College of Science, Imam Mohammad Ibn Saud Islamic University (IMSIU), Riyadh, Saudi Arabia; ^3^ Department of Biochemistry, Government College University Faisalabad, Faisalabad, Pakistan; ^4^ Department of Chemistry, Islamia College University Peshawar, Peshawar, Pakistan; ^5^ Department of Pharmaceutical Chemistry, Faculty of Pharmacy, Suleyman Demirel University, Isparta, Türkiye

**Keywords:** ultrasonic assisted approach, high yields, theophylline-1,2,4-triazoles, serine protease inhibitors, antibacterial agents, molecular docking, DFT study, SAR

## Abstract

Theophylline, a nitrogen-containing heterocycle, serves as a promising focal point for medicinal researchers aiming to create derivatives with diverse pharmacological applications. In this work, we present an improved synthetic method for a range of theophylline-1,2,4-triazole-S-linked N-phenyl acetamides (4a‒g) utilizing ultrasound-assisted synthetic approach. The objective was to assess the effectiveness of synthesized theophylline-1,2,4-triazoles (4a‒g) as inhibitors of HCV serine protease and as antibacterial agents against *B. subtilis* QB-928 and *E. coli* AB-274. Theophylline-1,2,4-triazoles were obtained in good to excellent yields (69%–95%) in a shorter time than conventional approach. 4-Chlorophenyl moiety containing theophylline-1,2,4-triazole 4c displayed significantly higher inhibitory activity against HCV serine protease enzyme (IC_50_ = 0.015 ± 0.25 mg) in comparison to ribavirin (IC_50_ = 0.165 ± 0.053 mg), but showed excellent binding affinity (−7.55 kcal/mol) with the active site of serine protease, better than compound 4c (−6.90 kcal/mol) as well as indole-based control compound 5 (−7.42 kcal/mol). In terms of percentage inhibition of serine protease, 2-chlorophenyl compound 4b showed the maximum percentage inhibition (86%), more than that of the 3,4-dichlorophenyl compound 4c (76%) and ribavirin (81%). 3,4-Dimethylphenyl-based theophylline-1,2,4-triazole 4g showed the lowest minimum inhibitory concentration (MIC = 0.28 ± 0.50 μg/mL) against the *B. subtilis* bacterial strain as compared to the standard drug penicillin (MIC = 1 ± 1.50 μg/mL). The other 4-methylphenyl theophylline-1,2,4-triazole 4e (MIC = 0.20 ± 0.08 μg/mL) displayed the most potent antibacterial potential against *E. coli* in comparison to the standard drug penicillin (MIC = 2.4 ± 1.00 μg/mL). Molecular docking studies further helped in an extensive understanding of all of the interactions between compounds and the enzyme active site, and DFT studies were also employed to gain insights into the molecular structure of the synthesized compounds. The results indicated that theophylline-linked triazole derivatives 4b and 4c showed promise as leading contenders in the fight against the HCV virus. Moreover, compounds 4e and 4g demonstrated potential as effective chemotherapeutic agents against *E. coli* and *B. subtilis*, respectively. To substantiate these findings, additional *in vivo* studies and clinical trials are imperative, laying the groundwork for their integration into future drug design and development.

## 1 Introduction

Hepatitis C virus (HCV) impacts over 3% of the global population, making it five times more prevalent than HIV (human immunodeficiency virus) infection (De Francesco et al., 2003; Arasappan et al., 2010). If left untreated, HCV infections can progress to cirrhosis, hepatocellular cancer, and liver failure (Bogen et al., 2006). Currently, there is no vaccine available to prevent HCV infection, and there is a lack of broadly effective therapy for individuals with HCV-associated chronic hepatitis (De Francesco and Carfí, 2007). The recommended treatments for HCV involve pegylated interferon-α (PEG IFN-α) injections and oral ribavirin. However, these treatments are only successful in achieving a persistent viral response in 50% of genotype-1 individuals and are associated with significant side effects such as hematological toxicities, flu-like symptoms, and neuropsychiatric events (Koev et al., 2007; Li et al., 2010). Consequently, there is considerable interest in discovering more potent therapeutics for the treatment of HCV infection (Pause et al., 2003; Xue et al., 2012).

**FIGURE 1:**
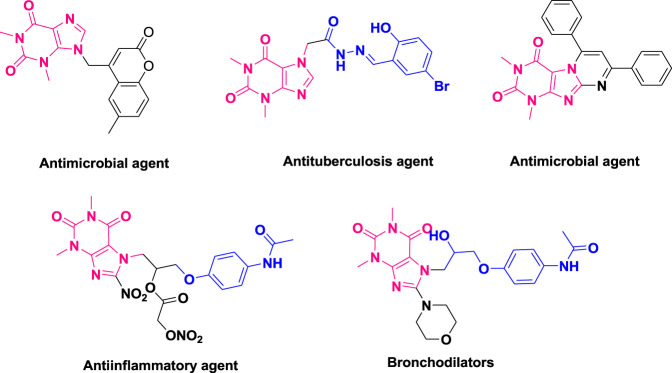
Reported biologically potent derivatives of theophylline.

Due to their substantial applications across industries, including biological, agricultural, medicinal and pharmacological fields, chemists have drawn their attention to various heterocyclic scaffolds, such as piperazines (Halimehjani et al., 2022), benzoxazole, benzothiazole (Zou et al., 2023), heterocyclic sulfonamides (Apiraksattayakul et al., 2022), benzofurans (Farhat et al., 2022), benzimidazoles (Nardi et al., 2023), thiadiazoles (Janowska et al., 2020), pyarzoles (Nazeer et al., 2023), lamivudines (Henriquez-Camacho et al., 2023), ciprofloxacin-based acetanilides (Zhang et al., 2018), triazoles (Irfan et al., 2024), quinoxalines (Montana et al., 2019), and oxadiazoles (Kerru et al., 2020; Irfan et al., 2022; Irfan et al., 2023). Notably, theophylline (Shahzadi et al., 2020; Shahzadi et al., 2022a), a naturally occurring methylxanthine, has found frequent therapeutic use in treating conditions such as asthma, respiratory deficits in premature infants, and chronic pulmonary obstructive disease (COPD). It also functions as a phosphodiesterase inhibitor (Ito et al., 2002; Saharan and Nantwi, 2006). Acefylline (Shahzadi et al., 2022b), a derivative of theophylline with pharmacological activity, serves various purposes, including bronchodilation, heart stimulation, diuresis, smooth muscle relaxation, antimicrobial and antituberculosis properties, and antiinflammatory effects (Foppoli et al., 2007; Mangasuli et al., 2018; Gopinatha et al., 2020; Shahzadi et al., 2020; Shahzadi et al., 2022a; Montaño et al., 2022), as illustrated in Figure 1.

**FIGURE 2:**
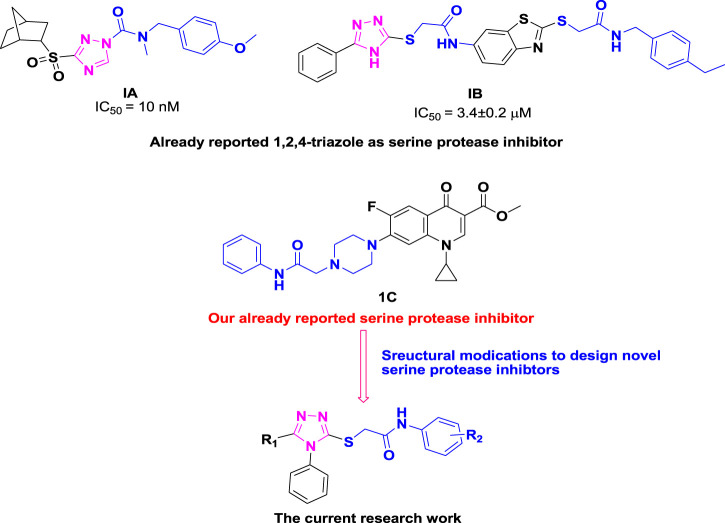
Current study rational design of theophyllines as serine protease inhibitors.

Similarly, heterocyclic scaffolds bearing the 1,2,4-triazole ring exhibit a broad range of pharmacological properties such as antihypertensive, antimalarial, antianxiety, antifungal, anticancer, antidiabetic, anticonvulsant and anti-tubercular (Abuo-Rahma et al., 2014; Mohassab et al., 2017; Montana et al., 2019; El-Sayed et al., 2020; Irfan et al., 2022; Irfan et al., 2023). 1,2,4-Triazole ring has been discovered to be essential component of serine protease inhibitor such as carbamoyl triazole **1A** and benzothiazole based triazole **1B**. Previously, our research group also identified ciprofloxacin-based acetanilide **1C** as serine protease inhibitor. In general, compounds containing the 1,2,4-triazole moiety exhibit good inhibitory effect against serine protease (Samanta et al., 2013; McConville et al., 2015; Akhtar et al., 2019; Kerru et al., 2020; Shahzadi et al., 2020; Shahzadi et al., 2022a) as depicted in Figure 2.

In a previous study, our research group outlined a traditional method for synthesizing theophylline-triazoles with moderate to good yields (66%–75%) (Ito et al., 2002). Subsequently, we have shifted our focus to enhancing reaction efficiency and yield by incorporating ultrasound irradiation to expedite the process. Building upon this and considering the importance of theophylline triazoles, our ongoing research involves the synthesis of *S*-alkylated *N-*aryl acetamide derivatives with a 1,2,4-triazole core under ultrasonic irradiation. We have undertaken this synthesis with the aim of evaluating the therapeutic potential of the resulting compounds against bacterial strains and as inhibitors of serine proteases.

## 2 Materials and methods

### 2.1 Materials

Ultrasound-assisted irradiation reactions were conducted using an ultrasound cleaner bath operating at a frequency of 47 kHz equipped with a mechanical timer and heater switch. All the chemicals, starting materials, and solvents utilized in these synthetic protocols were of analytical grade and procured from reputable suppliers Merck and Sigma Aldrich. The progression of the reaction and purity of compounds were monitored via thin layer chromatography (TLC) performed at silica gel plates. However, a UV lamp was used to visualize the spots on the TLC plate. Gallenkamp equipment was used to determine the melting points of target analogues. Spectral analysis including proton NMR (^1^H-NMR) at 400 MHz (δ = ppm) and carbon-13 NMR (^13^C-NMR) at 100 MHz (δ = ppm) was performed on a Bruker spectrophotometer.

### 2.2 Method of preparation

#### 2.2.1 Synthetic protocol for synthesis of theophylline/aryl-triazole hybrids (4a-h)

**SCHEME 1:**
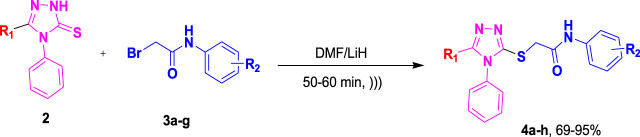
Synthetic pathway for synthesis of theophylline/aryl-triazole hybrids **4a-h**.

To the mixture of corresponding 1,2,4-triazole-3-thione (0.00037 mol, 1 equiv.) in 10 mL of DMF was added LiH (0.00074, 2 equiv.), and the obtained solution was irradiated by ultrasound over 15 min. Then, the mixture was subjected to 2-bromo-*N*-phenylacetamide **3a-g** (0.00037 mol, 1 equiv.) with further irradiation (Scheme 1). After reaction completion, as indicated by TLC, the reaction content was poured on crushed ice. Precipitates of the desired compounds were formed, which were recrystallized with ethanol. The characterization data of these synthetic molecules **4a–g** was in agreement with the previously published data (Batool et al., 2018; Shahzadi et al., 2021).

#### 2.2.2 Protease inhibition assay

The protease assay was carried out using a modified Kunitz caseinolytic assay. A test tube was filled with 800 μL of 1% casein dissolved in phosphate buffer at a pH of 7.5 and incubated for 10 min. Following the additive, 100 μL of TCA solution and 100 μL of sample were added. This reaction mixture will be placed in an incubator for 30 min before being filtered. After that, 50 μL of FC reagent and 312 μL of Na_2_CO_3_ solution were added to the solution mixture and incubated for 30 min. The optical decimal λmax = 660 nm was measured against a blank (Zhang et al., 2018).

#### 2.2.3 Antibacterial activity assay

The antibacterial therapeutic efficacy of each targeted compound was assessed using the disc diffusion method (Sarker et al., 2007; Siddiqa et al., 2018). A suspension containing 108 cfu/mL of bacteria per 100 μL was spread on to nutrient agar media using a sterilized loop. The molecules were dissolved in chloroform at a specific concentration (25 μg/100 μL) and infused into the sterilized filter paper discs (5.6 mm in diameter). Penicillin (25 μg/100 mL per disc) was used as a positive control. Following infusion of compound solution, these discs were positioned on agar plates inoculated with selected bacterial strains. After incubating plates at 27°C for 24 h, the inhibition zones (ZI) were measure in millimeters (including 5.6-mm disc diameter) in comparison with reference control drug.

### 2.3 Computational screening of the most bioactive triazole compounds

#### 2.3.1 Molecular docking analysis of the most bioactive compounds

For the computational studies against the NS3/4A protease of HCV, we utilized the protein structure having PDB ID: 6NZT (Meewan et al., 2019), while the structures of the compounds were prepared using the ChemDraw professional Ver-16 software. After structure preparation, the compounds were imported into the MOE (MOE Molecular Operating Environment, 2015) software, where the chemical structures were energy minimized, and then we proceeded with the preparation of the protein molecule, which was prepared by the structure preparation module of the MOE software, whereby all the missing atoms and the necessary hydrogen atoms were added to its structure. Finally, these compounds were docked with the protein, and their interactions with molecules were analyzed using the Biovia Discovery Studio (Mills, 2006) software.

#### 2.3.2 DFT studies of the most bioactive compounds

The DFT study was undertaken using the Gaussian program as reported before (Dennington and Keith, 2008; Muhammed and Aki-Yalcin, 2023). The resulting computation results were interpreted based on total energy, the highest occupied molecular orbital (HOMO) energy, and the lowest unoccupied molecular orbital (LUMO) energy obtained from the program, as well as computed parameters with respective formulas.

## 3 Results and discussion

### 3.1 Chemistry

As depicted in Scheme 1, ultrasound irradiation has been employed to enhance organic reactions in synthetic processes. Noteworthy characteristics of these methods include reduced costs, shorter reaction times, mild conditions, high yields, and an environmentally friendly and convenient methodology compared to traditional approaches. In a previous study (Shahzadi et al., 2021), our research group found that the conventional synthesis of acefylline-triazole resulted in yields ranging from 65% to 80%, requiring several hours to complete the reaction. To improve yield and reduce synthesis time, we utilized ultrasound-assisted techniques to achieve the same acefylline-triazole compounds.

Under ultrasound irradiation, compound **2** was reacted with 2-bromo-*N*-phenylacetamides **3a-h**, leading to the formation of *S*-alkylated analogues of triazole **4a-h** with good to excellent yields (69%–95%), as detailed in Table 1 (Batool et al., 2018). The ultrasound-assisted approach proved significantly more efficient, yielding substituted 1,2,4-triazole products at 69%–95% in a much shorter timeframe of 50–60 min as compared to the previously reported method, which required 24–48 h to furnish products at 65%–80% yield.

**TABLE 1:** Yields of theophylline–1,2,4-triazole analogues via ultrasound irradiation synthetic pathway.

Compounds	Conventional		Ultrasound-assisted		Found m.p. (^o^C)	Reported m.p. (^o^C) (Shahzadi et al., 2021)
	Method (Shahzadi et al., 2021)		Method (this work)			
	Yield (%)	Time (hours)	Yield (%)	Time (minutes)		
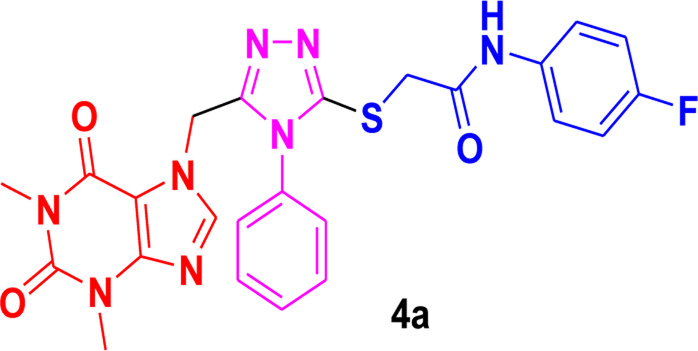	66	24–48	69	55	168	168
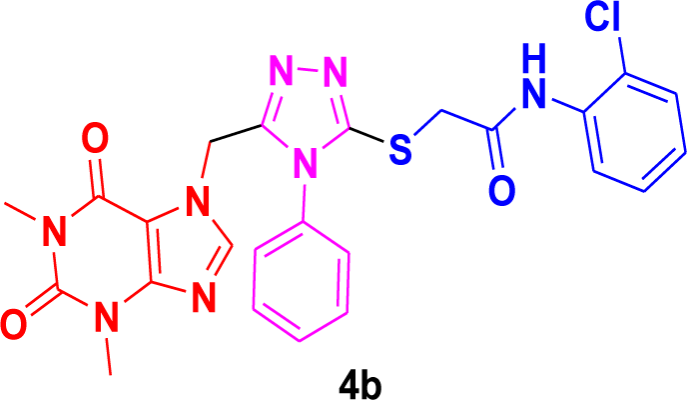	70	24–48	80	55	116–117	117
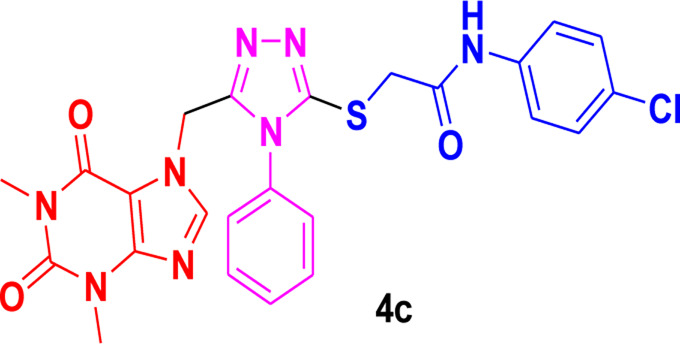	73	24–48	88	55	136	136
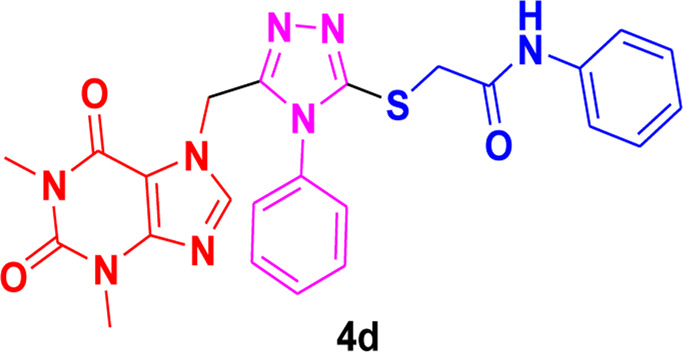	75	24–48	86	50	210	210
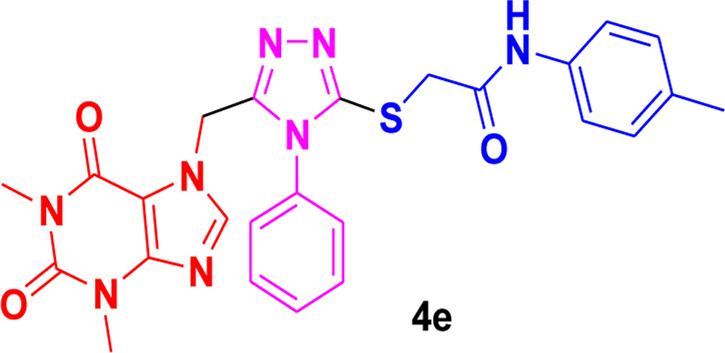	73	24–48	95	55	141–142	141
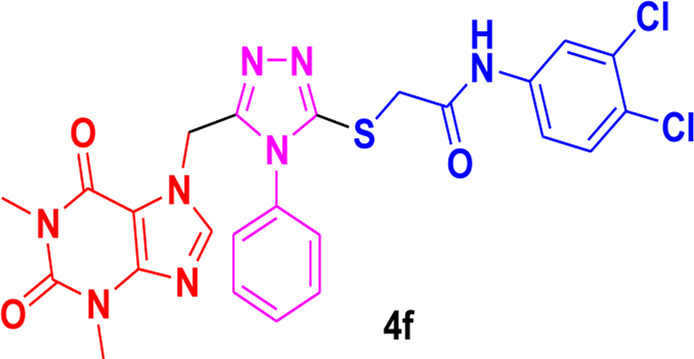	71	24–48	79	60	221–222	222
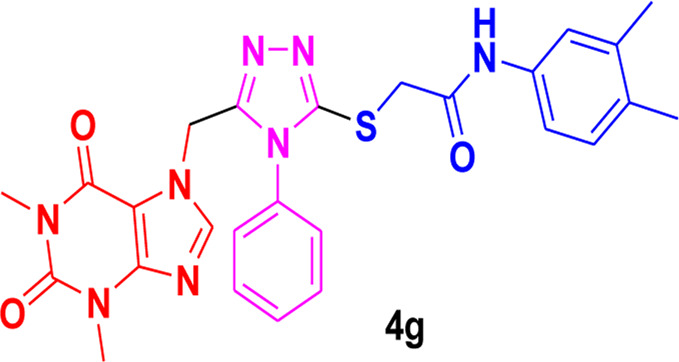	67	24–48	91	50	148–149	148

### 3.2 Serine protease inhibitory activity

The inhibitory potential of a series of synthesized triazole derivatives (4a–g) against serine protease was investigated. The results of their serine protease inhibition activity are summarized in Table 2. The IC_50_ values, indicative of the concentration required for 50% inhibition, revealed that all the designed triazole analogues exhibited noteworthy inhibitory potential (IC_50_ values = 0.015 ± 0.25 mg to 1.314 ± 0.00 mg) against serine protease in comparison to the reference standard drug ribavirin, with an IC_50_ value of 0.165 ± 0.053 mg.

**TABLE 2:** Theophylline-triazole molecules **4a-g** inhibitory activity against serine protease.

Molecules	% Age inhibitory activity (50 mM)	IC_50_ (mg)
**4a**	44	0.658 ± 0.00
**4b**	86	0.197 ± 0.00
**4c**	76	0.015 ± 0.25
**4d**	32	1.314 ± 0.00
**4e**	78	0.45 ± 0.00
**4f**	39	0.940 ± 0.50
**4g**	46	0.855 ± 0.25
**Ribavirin**	76.16	0.165 ± 0.053

Among the synthesized derivatives, compound **4c**, featuring an electron-withdrawing chloro group on the acetanilide aryl ring, demonstrated the most robust inhibitory activity against serine protease, boasting an IC_50_ value of 0.015 ± 0.25 mg. The second-most active molecule, **4b**, displayed an IC_50_ value of 0.197 ± 0.00 mg. Compounds **4a**, **4e**, and **4f** also exhibited promising inhibitory activity against serine protease, with IC_50_ values ranging from 0.203 ± 0.00 to 0.940 ± 0.50 mg. In contrast, compound **4d** was considered relatively less active, showing an IC_50_ value of 1.314 ± 0.00 mg. These findings underscore the potential of the synthesized triazole derivatives as effective inhibitors of serine protease, with compound **4c** standing out as particularly potent in this regard.

### 3.3 Structure-activity relationship of triazoles as serine protease inhibitory agents

The structure-activity relationship (SAR) was estimated for all the screen triazole molecules on the basis of nature of substituents as well as position of substituents on the aryl ring of *N*-phenylacetamide. It was shown that electron-donating and electron-withdrawing groups on the aryl ring of *N*-phenylacetamide increased the inhibitory potential as compared to the unsubstituted phenyl ring. The most potent hybrid in the series, compound **4c**, with the chloro group at the para position on the acetanilide aryl ring, was found to have an IC_50_ value of 0.015 ± 0.25 mg.

**FIGURE 3:**
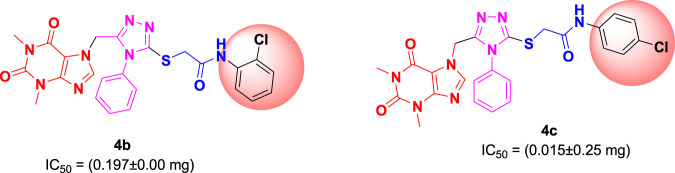
SAR of 1,2,4-triazole core based **4b** and**4c** derivatives.

However, changing the position of the chlorine atom from para to ortho in the acetanilide aryl ring decreased the activity as compared to **4c** such as compound **4b** (0.197 ± 0.00 mg). Furthermore, the placement of two chloro groups at meta as well as para positions further decreased the inhibitory potential of compound **4f** (0.940 ± 0.50 mg). Compound **4e** (0.45 ± 0.00 mg) bearing a methyl group at the para position exhibited significant activity, while the incorporation of another methyl group at the meta position slightly decreased the activity such as compound **4g** (0.855 ± 0.25 mg). Compound **4a** (0.658 ± 0.00 mg) having a mono-substituted para fluoro phenyl ring showed considerable activity. The least active compounds in the series were **4d** (1.314 ± 0.00 mg) which have an unsubstituted phenyl ring (Figure 3).

### 3.4 Antibacterial activity

Triazole-based compounds were analyzed for their *in vitro* efficacy against Gram-positive *B. subtilis* QB-928 and Gram-negative *E. coli* AB-274 bacterial strains via the disc diffusion method. Results predicted in Table 3 demonstrated that synthesized hybrids **4a–h** displayed moderate antibacterial potential. In particular, compounds **4e** and **4g** exhibited the most promising antibacterial potential values (0.20 ± 0.08 μg/mL and 0.28 ± 0.50 μg/mL) against *E. coli* and *B. subtilis,* respectively. The antibacterial effect of compound **4c** was observed to be similar in activity against *E. coli* (0.5 ± 0.00 μg/mL) and *B. subtilis* (0.5 ± 1.75 μg/mL), respectively. Compounds **4a**, **4d,** and **4f** showed lower antibacterial activity values, that is, 20 ± 2.25 μg/mL, 20 ± 1.25 μg/mL, and 18.48 ± 0.95, whereas compounds **4a**, and **4g** did not possess any antibacterial activity against *E. coli*. Compounds **4c** (0.5 ± 1.75 μg/mL) and **4e** (2.94 ± 0.06 μg/mL) when evaluated against *B. subtilis showed* moderate antibacterial activity, while compounds **4b**, **4d,** and **4f** did not provide any detectable results as shown in Table 3.

**TABLE 3:** Antibacterial therapeutic efficacy by measuring zone of inhibition.

Molecules	*Bacillus subtilis*		*Escherichia coli*	
	ZI (mm)	MIC (µg/mL)	ZI (mm)	MIC (µg/mL)
**4a**	7.75	20 ± 2.25	nd	nd
**4b**	Nd	nd	5	3.54 ± 1.34
**4c**	28.75	0.5 ± 1.75	15	0.5 ± 0.00
**4d**	Nd	nd	5	20 ± 1.25
**4e**	12.5	2.94 ± 0.06	20.5	0.20 ± 0.08
**4f**	Nd	nd	5	18.48 ± 0.95
**4g**	20	0.28 ± 0.50	nd	nd
**Penicillin**	18	1 ± 1.50	24	2.4 ± 1.00

### 3.5 Structure-activity relationship of the most potent triazoles as antibacterial agents

SAR (structure-activity relationship) of all synthesized compounds (4a–h) demonstrated that only compound **4e** having an electron-donating (CH_3_) substituent displayed the highest activity against *E. coli* (0.20 ± 0.08 μg/mL), whereas this hybrid possessed little activity against *B. subtilis* (2.94 ± 0.06 μg/mL). The compound **4g,** having a methyl group at the meta as well as para positions of the anilide ring, was the second-most potent hybrid of the series against *B. subtilis* (0.28 ± 0.50 μg/mL), but it was inactive against *E. coli*. The existence of a chloro substituent at the ortho position of the anilide ring as in compound **4b** (3.54 ± 1.34 μg/mL) and at the meta as well as para positions in compound **4f** (18.48 ± 0.95 μg/mL) decreased the antibacterial activity against *E. coli* and produced no detectable results towards *B. subtilis,* while compound **4d** (20 ± 1.25 μg/mL) having an unsubstitued anilide ring showed a similar effect. Placing a fluoro substituent at the para position of *N*-phenyl acetamide (4a) exhibited the least activity against *B. subtilis* (20 ± 2.25 μg/mL) and was found ineffective towards *E. coli*.

### 3.6 Molecular modeling studies of the acefylline derivatives for their binding affinity and conformational analysis with the HCV serine protease

Molecular modeling studies were conducted for the acefylline derivatives, which showed good anti-HCV serine protease inhibition activities in the *in vitro* experimental assays. We employed molecular docking techniques to identify the binding affinities and conformational bindings of these two acefylline derivatives (4b and 4c) to understand the inhibitory mechanism of the HCV viral serine protease, also known as the NS3/4A protease enzyme.

In these investigations, it was found that compounds **4b** and **4c** possess binding affinities of −7.55 and −6.90 kcal/mol with the active site of the HCV serine protease enzyme. The control drug, which is compound **5** (Meewan et al., 2019), is a small-molecule inhibitor of this serine protease enzyme. This compound **5** was able to show a binding affinity of −7.42 kcal/mol with this target enzyme, which, in comparison to our compounds, has a relatively similar binding affinity. Furthermore, analysis of the molecular interactions and binding conformations of compounds **4b** and **4c** revealed that these compounds show diverse types of molecular interactions with the active site of the target enzyme. It was found in the binding conformation analysis of these two compounds inside the serine protease active pocket that these compounds occupy the active site pocket and block access to the catalytic amino acids of this enzyme, which are key amino acid residues involved in the processing of the HCV viral proteins after infection with the HCV virus.

**FIGURE 4:**
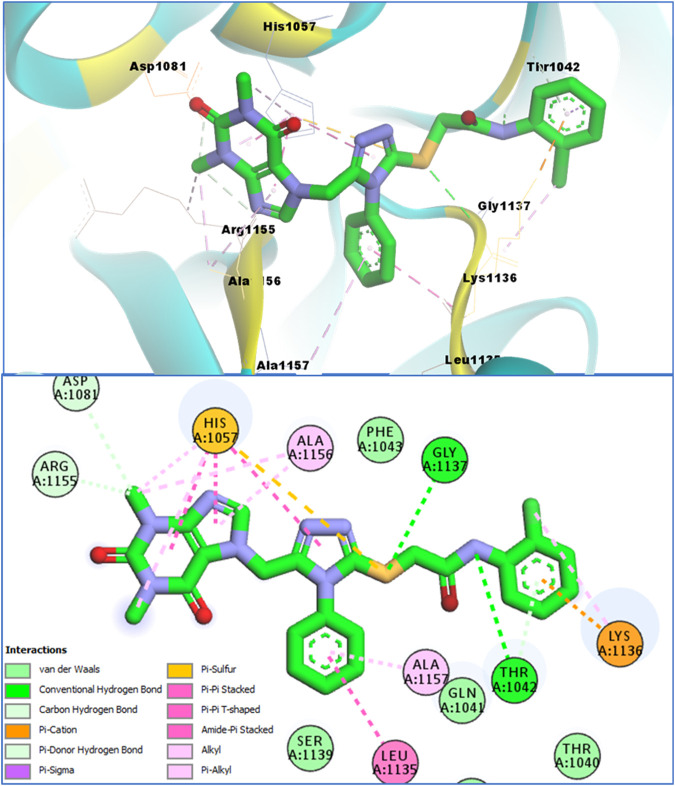
Conformational pose of 4b interacting with the NS3/4A protease of HCV.

It can be seen in Figure 4 that compound **4b** is in contact with the important ASP1081 and HIS1057 catalytic residues, engaging it via multiple types of molecular interactions. The acefylline moiety as well as the triazole scaffold of this compound can be seen interacting via diverse types of interactions, e.g., hydrogen bonds (both conventional as well as C-H hydrogen bonds along with Pi-Donor H-Bond). Other than these multiple types of hydrophobic interactions, which further stabilize a compound inside the active site of an enzyme like Alkyl, Pi-Alkyl Pi-Cation and Sigma, Pi-Sulfur, Amide-Pi Stacked, etc., other moieties of the **4b** compounds and the amino acid residues of the active site pocket of HCV serine protease can also be seen in Figure 4.

Similarly, the other compound **4c** was also able to show a similar type of conformation and molecular interaction with the target enzyme active site amino acid residues. The acefylline moiety of the **4c** was able to engage the important catalytic amino acid residues multiple times via conventional and carbon-hydrogen-type hydrogen bonding. The important SER1139 and HIS1057, which are the main catalytic amino acid residues, can be seen in Figure 4, making multiple strong interactions with the acefylline moiety of the **4c** compound. Apart from the acefylline moiety, other important scaffolds like the triazole and the other phenyl moieties can also be seen engaging in different types of molecular interactions with the important active site residues of this enzyme. Table 4 contains the binding energies and chemical structures of the investigated compounds with the target enzyme.

**TABLE 4:** Structures, binding affinities of the investigated compounds with the HCV NS3/4A Protease.

Structures of bioactive	Binding affinity with HCV serine protease
1,2,4-Triazole	
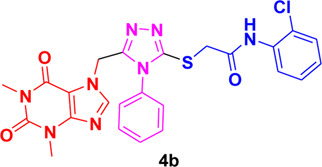	−7.55 kcal/mol
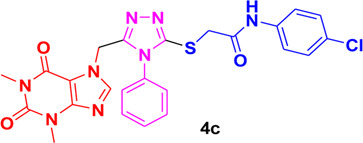	−6.90 kcal/mol
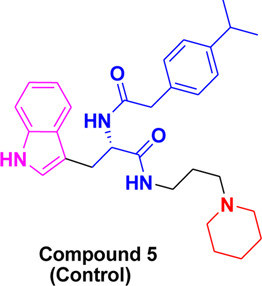	−7.42 kcal/mol

### 3.7 DFT studies

The HOMO, LUMO and total energies of the relatively active synthesized compounds were obtained from the DFT computation. Then, the other related parameters were calculated with the respective formulas (Table 5) (Zeyrek et al., 2021; Arslan et al., 2023).

**TABLE 5:** Computed energy values from the DFT computation (in eV).

Parameters	4b	4c	4e	4g
E_total_	−43,961.620	−42,892.526	−44,641.183	−45,710.821
E_HOMO_	−6.482	−6.447	−6.072	−5.965
E_LUMO_	−1.711	−1.696	−1.654	−1.657
ΔE	4.771	4.751	4.418	4.308
Ionization potential (IP = -E_HOMO_)	6.482	6.447	6.072	5.965
Electron affinity (A = -E_LUMO_)	1.711	1.696	1.654	1.657
Chemical potential (µ = -(I + A)/2)	−4.097	−4.072	−3.863	−3.811
Hardness (η = (I-A)/2)	2.386	2.376	2.209	2.154
Mulliken electronegativity (ᵡ = (I + A)/2) (Parr et al., 1978)	4.097	4.072	3.863	3.811
Softness (S = 1/2η)	0.209	0.210	0.226	0.232
Electrophilicity index (ꞷ = µ^2^/2η) (Chattaraj et al., 2006)	3.508	3.482	3.373	3.369
Maximum charge transfer (ΔN_max_ = (I + A)/2(I-A)) (Koopmans, 1934)	0.859	0.857	0.874	0.885

The DFT analysis showed that all the investigated compounds had similar electrochemical values with slim differences. As HOMO represents electron donors and LUMO represents electron acceptors of a molecule, they are utilized to predict electron exchange capacity (Miar et al., 2021). According to predictions, **4g** had the highest HOMO energy value, while **4b** had the lowest. The HOMO energy value of **4g** was predicted to be the highest whereas **4b** had the lowest value. Similarly, **4e** had the highest LUMO value whereas **4b** had the lowest value (Table 5). Hence, **4g** is expected to give its electrons relatively easy whereas **4e** is expected to accept electrons with relatively higher affinity. HOMO-LUMO energy gaps of molecules give clue about the relative stability of them. In general, higher energy gap manifests higher chemical stability (Ruiz-Morales, 2002). In this study, the greatest energy gap among the tested compounds was produced by compound **4b** (Table 5). Hence, **4b** is anticipated to display the highest chemical stability. Global hardness displays resistance of atoms to electron transfer. The DFT computation revealed that **4b** had the highest global hardness value as expected. This study implicated that **4b** might be the most stable as well the least reactive (Han et al., 2022).

**FIGURE 5:**
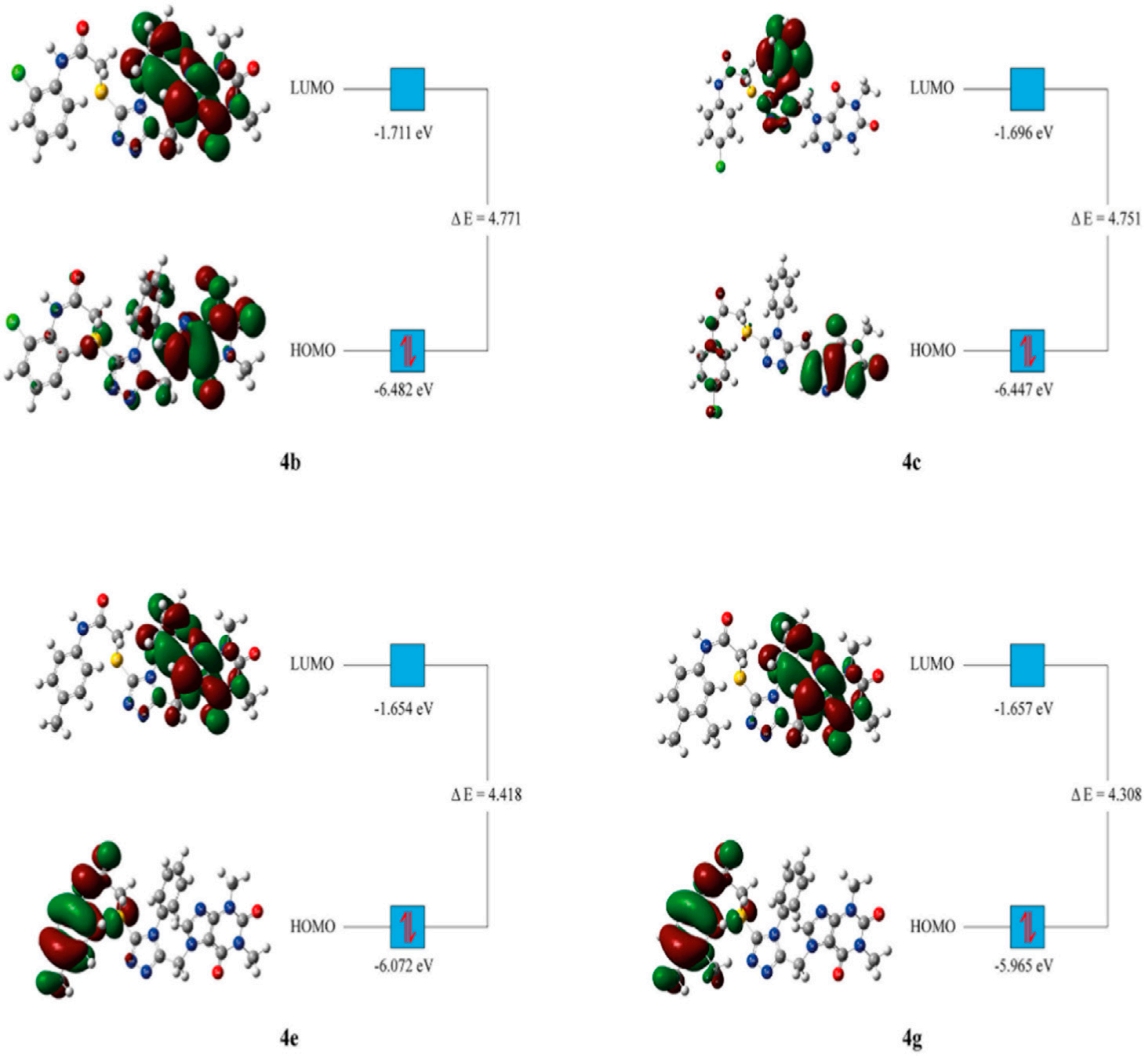
HOMO-LUMO orbitals obtained from the DFT computation.

HOMO-LUMO orbital orientations were similar for some derivatives and different for some others. In this regard, orbital orientations of **4e** and **4g** were similar with each other. HOMO orbitals were predominantly observed around the *p*-tolyl acetamide group and sulfur next to it (Figure 5). Similarly, LUMO orbitals were mainly observed around the purine heterocyclic group and its substituents for both of them. In addition to this, some LUMO orbitals were observed around the triazole ring (Figure 5). However, HOMO and LUMO orbitals of compound **4b** were mainly concentrated around the purine heterocyclic group and also sparsely located around the triazole ring. Furthermore, LUMO orbitals were observed around the sulfur of compound **4b**. Orbital distribution of **4c** was different from the other compounds. The LUMO orbitals were concentrated mainly around the triazole ring and its phenyl substituent. On the other hand, the HOMO orbital distribution was concentrated around the purine ring and some more orbitals were also observed around 4-chlorophenyl group next to the acetamide functional group (Figure 5).

## 4 Conclusion

The synthesis of theophylline-linked 1,2,4-triazole compounds (4a‒g) was achieved with excellent yield under ultrasonic irradiation, surpassing the conventional approach’s reported yield of 66%–75%. The ultrasonic-assisted method demonstrated advantages such as shorter reaction times and higher yields of 1,2,4-triazole products (69%–95%). Notably, among the synthesized structural hybrids, the 1,2,4-triazole compound **4c** (IC_50_ = 0.015 ± 0.25 mg), featuring a 4-chlorophenyl ring, exhibited superior serine protease inhibitory activity compared to the standard drug ribavirin (IC_50_ = 0.165 ± 0.053 mg). Molecular docking studies revealed that triazole compound **4b** exhibited a stronger binding affinity score than **4c** and the control drug **5** with the active site of serine protease enzyme. DFT study results were consistent with *in vitro* and molecular docking findings. In terms of inhibition, compound **4b** demonstrated higher inhibition (86%) of serine protease compared to 3,4-dichlorophenyl compound **4c** (76%) and the standard drug ribavirin (81%). Thus, compounds **4b** and **4c** emerged as more promising serine protease inhibitors than the standard drug, ribavirin.

Structure-activity relationship (SAR) analysis revealed that nature of substituents as well as position of substituents on the aryl ring of *N*-phenylacetamide played a crucial role in protease inhibition. Additionally, the synthesized compounds exhibited significant antibacterial potential against *Bacillus subtilis* and *Escherichia coli*. Theophylline-1,2,4-triazole **4g**, based on 3,4-dimethylphenyl, showed the lowest minimum inhibitory concentration (MIC = 0.28 ± 0.50 μg/mL) against *B. subtilis* compared to the standard drug penicillin (MIC = 1 ± 1.50 μg/mL). Another compound, **4e**, featuring 4-methylphenyl theophylline-1,2,4-triazole, displayed the most potent antibacterial potential against *E. coli* (MIC = 0.20 ± 0.08 μg/mL) compared to penicillin (MIC = 2.4 ± 1.00 μg/mL). In conclusion, this study suggests that modifications in triazole hybrids could lead to the development of more effective antibacterial and serine protease inhibitor compounds.

## Data Availability

The original contributions presented in the study are included in the article/Supplementary Material, further inquiries can be directed to the corresponding authors.
